# A Survey for the Ranking of Trajectory Prediction Algorithms on Ubiquitous Wireless Sensors

**DOI:** 10.3390/s20226495

**Published:** 2020-11-13

**Authors:** Muhammad Daud Kamal, Ali Tahir, Muhammad Babar Kamal, Faisal Moeen, M. Asif Naeem

**Affiliations:** 1 Institute of Geographical Information Systems, National University of Sciences and Technology, Islamabad 44000, Pakistan; mkamal.ms15igis@igis.nust.edu.pk; 2 Department of Computer Science, COMSATS University, Islamabad 44000, Pakistan; sp18-rcs-027@student.comsats.edu.pk; 3 Department of Computer & Decision Engineering (CoDE), Université Libre de Bruxelles, 1050 Bruxelles, Belgium; ofaisal@ulb.ac.be; 4 Department of Computer Science, National University of Computer and Emerging Sciences (NUCES), Islamabad 44000, Pakistan; asif.naeem@nu.edu.pk or; 5 School of Engineering, Computer & Mathematical Sciences, Auckland University of Technology, Auckland 1010, New Zealand

**Keywords:** wireless sensors, global positioning system (GPS), prediction algorithm, Markov model, hidden Markov model, T-pattern tree, Bayesian networks, trajectories survey, indoor navigation, outdoor navigation

## Abstract

The number of wireless sensors in use—for example, the global positioning system (GPS) intelligent sensor—has increased in recent years. These intelligent sensors generate a vast amount of spatiotemporal data, which can assist in finding patterns of movements. These movement patterns can be used to predict the future location of moving objects; for example, the movement of an emergency vehicle can be predicted for health care decision-making. Although there is a body of research work regarding motion trajectory prediction, there are no guidelines for choosing algorithms best suited for individual needs in uncertain and complex situations and as per the application domains. In this paper, we surveyed existing trajectory prediction algorithms. These algorithms are further ranked scientifically in terms of accuracy (performance), ease of use, and best fit as per the available datasets. Our results show three top algorithms, namely NextPlace, the Markov model, and the hidden Markov model. This study can be beneficial for multicriteria decision-making for various disciplines, including health care.

## 1. Introduction

Movement analysis and prediction have gained much attention in recent years. Knowing a future location in advance can assist in future predictive planning, give a strategic advantage, and create a perception of the unknown. With the availability of intelligent sensors such as the global positioning system (GPS), communities are becoming ubiquitous. Simultaneously, many application domains including health care are becoming more popular. Over the years, numerous trajectory prediction algorithms have been proposed. The focus of this study is to survey the available algorithms and offer a methodology for ranking the algorithms in terms of efficiency, performance, and ease of use. Broadly, trajectory prediction algorithms are derived from machine learning approaches such as Bayesian networks [1], hidden Markov models [2], decision trees [3], neural networks [4], and state predictor methods [5].

This survey paper aims to assist researchers and the industry in selecting the algorithms for future location prediction. In the existing surveys, however, there are no proper guidelines for algorithm selection as per the probable use case. One such survey is [6], in which the authors used five algorithms for four users with different patterns. In most of the studies, a few aspects of trajectory prediction are discussed, and nearly all examples relate to studies that focus on indoor and outdoor navigation. In some studies, the authors have validated the accuracy of these algorithms on given datasets. In our review, we investigated 28 algorithms mostly used in trajectory prediction.

Here, we present a comprehensive survey of several algorithms. The survey starts with a discussion on public and proprietary datasets. We designed our methodology for ranking of these algorithms based on the number of citations, type of dataset, and accuracy. Subsequently, when the rankings were created, the paper was categorized based on machine learning approaches. For example, for each approach, we investigated the type of algorithm, accuracy, dataset, setting (indoor or outdoor), and the number of citations. A detailed discussion on each approach was incorporated for better insight. Finally, the top three algorithms, which were NextPlace [7], the Markov model [8], and the hidden Markov model [9], were proposed.

This research describes the selected studies and discuss the results of these trajectory prediction algorithms highlighted in the literature. This information will benefit the readers in terms of providing apparent aspects regarding the characteristics of these algorithms. The outcomes featured the qualities and shortcomings of the algorithms best studied; they will fill in as a helpful guide to the research community as well as the industry in understanding which techniques to utilize. Thus, developers will hugely benefit from this in terms of not being dependent on numerous case studies available on the Internet.

The remainder of this paper is structured as follows: In Section 2, a summary of the existing trajectory prediction algorithms is given. In Section 3, the related literature review is described. The available online datasets are presented in Section 4, while public and propriety datasets and their rankings are included in Section 5. The methodology is presented in Section 6, and the results of our survey are presented in Section 7. Finally, conclusions are given in Section 8.

## 2. Existing Trajectory Prediction Algorithms

This section presents all the unique algorithms being proposed by researchers.

### 2.1. Markov Model

The Markov model [8,10] is known for randomly changing systems. This model describes a sequence of probable events, whereas the probability of each state depends on the previous event state. In terms of trajectory prediction, this model gives a probabilistic forecast for future locations in light of previous segments of a given road network. The Markov model gives a probabilistic prediction over future segments based on past segments. The standard, first order Markov model says that the probability distribution **P**, if $X_{1}$ for the next segment is independent of all but $X_{0}$, for the current road segment is as follows:

$$P\left(X_{1}\;|X_{0},\;X_{1},\;X_{2},\ldots X_{n}\right)=P\left(X_{1}|X_{0}\left(1\right)\right).$$

### 2.2. Hidden Markov Model

The hidden Markov model (HMM) is a system that is modeled and considered as a Markov process [11]. The HMM is a random process in which the future does not depend on the past but only the present state. The HMM, the Markov process under consideration, has hidden states. The HMM is extensively used in many trajectory prediction algorithms, especially when finding the histories of vehicle trajectories.

Let $Y_{n}$ and $X_{n}$ be stochastic processes in discrete time and n≥1. The pair is $\left(X_{n},Y_{n}\right)$ in the hidden Markov model if

$X_{n}$ is the Markov process and is (hidden) directly not observable;

$$P(Y_{n}\in A\;|\;X_{1}=x_{1},\ldots ,X_{n}=x_{n})=P(Y_{n}\in A\;|\;X_{n}=x_{n})$$

for every n≥1 where { $x_{1},\ldots ,x_{n},$} and a measurable arbitrary set of *A*.

### 2.3. T-pattern Tree

The T-pattern tree is used to extract the periodic activity patterns from the frequently visited places [12]. In a typical tree structure, the nodes represent frequently visited places while the edges represent regions w.r.t travel time. The path of the tree is calculated based on the common prefix of T-patterns. For trajectory prediction, a T-pattern tree is being efficiently used when the algorithm is studying moving objects. The T-patterns are mixed in an already existing tree called the T-pattern tree. Nodes of the tree are areas that were periodically visited and the points highlight the traveling among regions which is donated along with the travel time. Each of the known T-patterns ends up in a known path on the tree segment. This tree might be referred as a global model of the actual mobility information, which represents every single infrequent trajectory once amplified with a default path.

### 2.4. Bayesian Network

The Bayesian network is a statistical technique that is used to support decision-making while taking uncertainty into account [13]. It uses a directed acyclic graph where each node represents a random variable with a finite set of mutually exclusive states. The trajectory information (such as spatiotemporal sequences, regions, and traversal paths) can be modeled easily into a Bayesian network. A Bayesian system is a portrayal of irregular factors on a graph and demonstrates the reliance conditions of arbitrary factors by methods for a directed acyclic graph (DAG). Bayesian networks are DAGs whose focus is to highlight the unpredictable elements or factors in the Bayesian sense: they may be unmistakable sums, inactive components, hidden parameters, or hypotheses. Edges represent contingent conditions; points that are not associated (there is no way from one variable to the next in a Bayesian system) represent a variable that is restrictively autonomous of each other. Every point is related to a likelihood work that takes, as info, a specific arrangement of qualities for the point’s parent factors. It gives (as yield) the likelihood (or likelihood dissemination) of the variable represented by the point. Let data be *i* and let θ be the parameter in Bayesian probality where posterior probablity p(θ∣i)∝p(i∣θ)p(θ) is computed by likelihood p(i∣θ) and prior probability p(θ).

(1) p(θ,α∣i)∝p(i∣θ)p(θ∣α)p(α))

### 2.5. Apriori-Traj Algorithm

The Apriori algorithm has been used for trajectory prediction based on frequently occurring sequences; the Apriori-Traj algorithm is the modified version which combines movement rules from moving objects database [14]. The movement rules are formed by splitting trajectories into sub-trajectories with a certain level of confidence. The Apriori-Traj algorithm was proved to be a good trajectory prediction algorithm which also provides simplification as one of the generalization techniques when the data size is large. In the Apriori-Traj algorithm initially, all regular trajectories of the length 1 (i.e., every single successive edge) are found. It links nearby continuous edges frame applicant trajectories of length 2. Next, arrangements of continuous trajectories of the length k in light of successive trajectories of the length (k − 1) are discovered iteratively. In every cycle, an agreement of k-component competitor trajectories were framed by joining covering numerous trajectories. Competitor trajectories were not checked in this algorithm for the regulation of rare sub-trajectories on the grounds that the strategy does not deliver any pointless competitor trajectories.

### 2.6. Traj-Prefix-Span Algorithm

The Traj-Prefix-Span algorithm is a modified version of the Prefix Span Algorithm [15]. The algorithm works in three phases. Firstly, it performs the full scan of the trajectory database in order to discover frequent trajectories. Secondly, each frequent trajectory is projected to create a projected trajectory database. The final step involves recursion to further project the database until the desired results are obtained. An information mining approach was created to address the issue of anticipating the area of a mobile device. Databases of moving entities are mined for areas to discover frequent trajectories and development orders. At that point, the direction is coordinated for moving the objects with the database of development principles to construct a probabilistic model of device’s location.

### 2.7. Query Triggered Revision

Query Triggered Revision (QTR) works to address the problem of Speed Update Triggered Revision (SUTR) [16]. In a moving object scenario, frequent travel speeds are obtained from GPS sensors. To predict the future speed at a given interval, all trajectories need an update. This process can be computationally very challenging, and performance issues may arise when massive trajectory data are processed. QTR provides a mechanism to update the trajectory database only when needed (query is executed, for example, range query). In Query Triggered Revision (QTR), rather than reexamining trajectories for each speed refresh, it amends them just while responding towards a query. For point inquiries, QTR functions when a point query is collected at the time ‘t’. The trajectory of the inquired object, which is indicated as Tr, is recovered from the database. The potent path that is followed ‘t’ on the course of Tr is perceived where the future rates for these squares are predicted. After that Tr is amended in view of the predicted velocities. In the end, the reconsidered Tr is utilized to answer the query.

### 2.8. Hybrid Genetic Algorithm

Hybrid Genetic Algorithm (HGA) is an extension of the Genetic Algorithm which can solve multiple problems, including vehicle routing [16]. The algorithm typically works on population size, iteration number, the crossover rate, and the mutation rate. Furthermore HGA is further classified into HGA1 and HGA2 which are two techniques to generate a population of initial chromosomes. Overall the HGA algorithm is efficiently used to solve efficient vehicle routing, which can be related to trajectories. Hybrid Genetic Algorithm is a stochastic streamlining system that performs cycles by applying Genetic algorithms operators (selection, mutation, crossover) to the point when some coveted union criteria are met. The initial idea of a genetic algorithm is considered to maintain a population of candidate solutions that evolves under selective pressure. The Hybrid genetic algorithm produces the beginning chromosomes of the issue, after the genetic algorithm parameters, that are: iteration number, the crossover rate, the population size, and the mutation rate are set. If there are i pots then each chromosome occupies i links. The routing represents for each link together with the delivery sequence of vehicles for a particular lot.

### 2.9. Seman-Predict

Semantics within trajectory has gained much attention in the recent past [17]. The algorithm assigns semantic tags to sequences. These tags can identify the specific activities at the given locations. The trajectory next location prediction is then made by using visited semantic locations. This algorithm is further divided into the on-line prediction and off-line mining. Overall the algorithm has achieved excellent performance. For capturing the landmarks, a semantic trajectory has a sequence of locations tagged with semantic tags known as semantic locations. These location-based semantic tags infer the exercises being completed in the trajectory. Trajectories are labeled with various semantic tags, for example, School, Stop, and so on. Here location prediction given the semantic trajectories of portable clients are assessed utilizing a strategy that comprises of two noteworthy modules: (1) Off-line mining module, and (2) On-line prediction module.

### 2.10. The Hybrid Prediction Model

The Hybrid Prediction Model algorithm provides an accurate prediction for near and distance-time queries [18]. The algorithm works on moving object pattern information and motion function. The algorithm also takes into account indexing for fast information retrieval. The algorithm has proved efficient on reasonable data sets. This model manages the prediction of where a specific item will be situated later on as well as objects mobility patterns and also the object’s latest location. Thus, the trajectory pattern of the object is found, and after that, an entrance technique is utilized for ordering the productive inquiry process. Two different methods are adopted for querying. For near off time inquiries, Forward Query Processing is utilized, which treats the recent trajectory of an object as an essential parameter for predicting the near future locations. Since past trajectories turn out to be not so useful for prediction, the Backward Query Processing is utilized. For rankings of the pattern selection, this procedure appoints lower loads to commence comparability measures and higher loads to results that are closer with the request time.

### 2.11. State Predictor Method

The State Predictor Method works on branch prediction techniques [19]. These techniques are known from high-performance processors that are adapted to context prediction. The authors proposed a one-level two-state predictor and local two-level context predictor with 2-state predictors in the 2nd level. This algorithm is being used for person tracking as a real movement object use case. The state prediction model utilizes measurable methods to anticipate the state of an occasion in the future, paying little attention to when it actually happened. Essentially it is used to foresee results. Future location prediction in State Predictor Method is advanced from branch prediction systems of current superior microprocessors. In the first place, the 2-bit branch predictor depends on the 1-level 2-state predictor or briefing the 2-state predictor. Analogically, the 2-state predictor holds two states for each conceivable next setting of the branch predictor; a weak and a strong state. For whatever length of time that the prediction is right, the predictor remains in the strong state mentioned as C(1). If the individual changes from setting X in another setting unequal C (e.g., Z), the predictor switches in the weak state C(0) prediction still set to C. On the off chance that the predictor is in a weak state (e.g., C(0)) and misses once more, by then, the predictor is set to the feeble condition of the new setting which will be foreseen next time. The 2-state predictor can be proficiently stretched out by new states amid run-time. n→numberofstates:(n>2),k→barrier:1≤k≤n

(2) s≥k:supplypredictionresult

(3) s<k:detainpredictionresult

### 2.12. Trajectory Similarity-Based Approach for Location Prediction (TLP)

The TLP algorithm works on the social mobility paradigm. The group of users are discovered having high trajectory similarity [20]. TLP is a multi-step process. In the first phase, the user personal mobility model is built. Then a smaller subset of data is derived from the more massive datasets. In the last step, mobility models are applied to the group based on similarity in order to predict the locations. Trajectory similarity-based location prediction utilizes social contagion theory and a similarity figuring based trajectory strategy alongside the trajectory examining, which quickens the way towards processing the similitudes. Initially, TLP accumulates all clients’ mobility information and moves the information into each client’s trajectory Traj. Second, it filters Traj to get the state set X, Y, and after that computes the state exchange matrix $P_{i}$. Third, TLP exploits covering calculation to separate the client’s consistency subset *U* sub. Moreover, it processes the similarity between Traj *i* and each client’s trajectory and gets clients close to personal trajectory similarity to assemble $C_{i}$. At long last, TLP consolidates the state trade network $P_{i}$ and others who are in $C_{i}$ and receives the client’s area forecast model *P* to predict.

(4) $$P_{predict}^{\left(i\right)}=a\sum_{U_{j}\;\epsilon \;C_{i}}P_{j}+\beta \;P_{i}$$

### 2.13. Gaussian Process Regression (GPR)

GPR is being used for prediction of spatiotemporal activities across many location-based services applications [21]. In this algorithm, a hidden dependant relationship in spatiotemporal sequences is modeled as a Gaussian process. GPR can be visualized as a distribution function to predict mobility. Overall, the GPR has achieved higher prediction accuracy. Gaussian process regression is a procedure by which an arrangement of values is introduced, and after that, those values are demonstrated by a Gaussian process which relies upon co-variances that happened before the present set of values. Initially, GPR separates and measures the hidden spatial and temporal qualities of the information. Next, it deliberately investigates the causality connection between these idle highlights and human spatial-fleeting exercises. Lastly, the relationship between them is demonstrated numerically for future prediction.

(5) $$f\left(a\right)\underset{}{\to }Gaussian\_Process\left(mean\left(a\right),convergence\left(a,a^{\prime }\right)\right)$$

## 3. Related Work

This section provides a brief on available algorithms in the literature. In a survey carried by [22], the authors emphasized the importance of mobile wireless systems in location prediction. The authors briefly described different types of location prediction and analysis algorithms. These algorithms are further categorized into domain-independent algorithms and domain-specific algorithms. The focus of their research was very limited to location prediction algorithms.

In a different study, the authors focused on the improvement of safety on the road by performing a survey of ways. The idea was to avoid hazardous accidents by predicting such situations in advance to practice safety precautions [23]. The authors based the study on the models that describe motion, risk, and further describing how the single motion model effects the selection of the estimated method. They used different simulation models such as Monte Carlo, physics-based motion models, dynamic models, kinematic models, Gaussian noise simulation, and maneuver-based and interaction-aware models which are built on dynamic Bayesian networks.

In another survey [24], issues regarding the mobility of different individuals were studied, such as transportation mode, patterns of trajectory, the significance of location, and other location-based models. The authors performed a detailed review of many different algorithms, techniques, and comparing alongside the obtained results with the issues of an individual’s mobility. Two types of graph approaches were used for mining trajectories from raw traces—first being the transitions among critical scenarios, and second being that the trajectories are changed to a spatiotemporal sequence.

The author used Augsburg indoor location tracking benchmarks as predictor loads and also used various techniques to model activities. The accuracy of different prediction methods was investigated and different location trajectories that were most frequently visited are studied [6]. The scenarios involved visiting various offices in the building area under study. These techniques were studied, including Bayesian networks, neural networks, state, and Markov predictors. The authors established the fact that there were individual variations in the accuracies of different predictive models.

In a similar survey, authors study regarding the spatial-temporal context of individuals visits [25]. Both spatial, historical trajectories, as well as temporal periodic patterns, are considered, and improvement in the current approaches has been achieved by the use of smoothing techniques on both patterns. The authors studied and reviewed nine baseline models to evaluate the model that they proposed which include Hierarchical Pitman-Yor (HPY) prior model, HPY Prior hourly model, HPY prior daily model, Most frequently visit model, order-1 Markov model, fall-back Markov model, most frequently hourly model, most frequently daily model, and most frequent hour-day model. According to their study, the model owed its improved structure to the pre-existing approaches.

Authors in [26] focused on developing a probabilistic model based on the generations of trajectories by tracking different objects over time. The survey also emphasizes on the use of topographical maps to specify the points of interest which are, in turn, connected by activity paths that explain the way how the object’s motion pattern takes place. The main aim of the model is to concentrate on the events of interest. This way, the surveillance systems automatically focus on events such as abnormality detection, prediction of activities, the interaction of objects, online activity analysis, classification of a path, speed profiling, and virtual fencing.

Similarly, the conventional methods were surveyed for the automotive industry and the advancement in the risk assessment techniques for intelligent vehicles [27]. They classified the motion and risk of vehicles based on semantics related to movement. The results showed that the choice of risk estimated method along with the motion model are the two major components for motion prediction and risk estimation for intelligent automobiles. Similarly, a study analyzed the different computational approaches developed for personal mobility [28]. An experimental analysis was performed on the unpublished mobility data of 15 users in the Helsinki metropolitan area. A variety of existing personal mobility methods were analyzed and the performance of such methods were evaluated. They also categorized the evaluation criteria to differentiate between the evaluation measures.

In a similar study [29], the authors reviewed the existing solutions for Geolocation Prediction (GP) and divided geolocation prediction into two primary parts. The initial steps proposed manufacturing Mining Popular Geolocation Region (MPGR), and second is Mining Personal Trajectory (MPT). The results described the basic concepts of GP, the characteristics of MPGR and MPT. They also discussed the limitations, openings, and the future geolocation prediction analytical trends for mobility big data.

In order to extend our discussion, we have described spatiotemporal approaches for their effectiveness, performance, and effortless use for predicting the future locations. One such studies is a model based on the spatiotemporal context of the user visiting history proposed by [25] for location prediction. The authors illustrated the behavior of historical spatial and temporal trails from user’s travel patterns. By applying smoothing methods to the model training, the authors obtained significant improvement in comparison to other similar approaches.

An algorithm which encompasses the trajectory patterns of a single user was developed [30]. The data were collected for four months and used to extract location from the data. For creating a predictive model of the user’s trajectories, these locations were considered. The authors suggested that these methods might be able to find sites that are substantially meaningful to a particular user.

Bayes-based predictors were used to add to the performance of their prediction for leveraging big data [31]. They studied a large Caller Data Record (CDR) dataset. Initially, they explored the dataset and found that they can use call activity to generate prior probabilities for use in a Bayes predictor; this was the baseline reason for which the authors developed an enhanced Bayes predictor, which uses a distance threshold and users’ regular location to improve the generation of their probabilities. Bayes predictor increased by 17 percent after enhancements proposed the experimental result. As per their concluding results, it was inferred that it is attainable to help massive cellular data to increase location predictors without depending on extraneous data.

A hybrid system was proposed by [32] for the location identification and prediction of the critical issues of location-based facilities. They used a hybrid method combining *k*-nearest neighbor (*k*-NN) with a decision tree to effectively recognize the sites in both indoor and outdoor environments. For the location prediction framework’s part, the hidden Markov model (HMM) was utilized to identify the client’s next location. The location grouping together with other contextual data.A probabilistic model decreases the complexity, the quality of developing, and restrains time of the execution. G-implies calculation was utilized for the proposed framework, which performs just on the former pattern of points. The accuracy execution was assessed for the expectation display on cell phones. The authors achieved prediction accuracy higher than 90 percent through these experiments. Similarly, an algorithm is developed for next location prediction for mobility modeling of a single user called a multi-order Markov chain (n-MMC) that keeps track of the n previous visited places [33]. The prediction algorithm achieved a 70 to 95 percent accuracy for three different datasets. The authors proposed a mining algorithm on the client’s mobility patterns and helped to predict the next location. They used simulation to evaluate the performance of the algorithm.

Furthermore, two other prediction algorithms were used by [34] called Mobility Prediction based on Transition Matrix (TM) and Ignorant Prediction. Similarly, a model is presented for trajectory pattern discovery for objects that are actively moving [14]. A movement rule gives a generalized view of a large set of moving objects and allows prediction of the next location of a moving object. Moreover, a technique is developed to predict the next place of a moving object. For a moving object, the prediction of the future location depends on the past developments of every single moving item in a specific range without taking into consideration any data about the user [12]. The beforehand proposed systems utilized the transient data in order to arrange occasions. The T-pattern was utilized, which considers temporal dimensions.

A future location prediction method, i.e., Spatial-Temporal Recurrent Neural Networks (ST-RNN) was presented by [20]. The experimental results on real datasets showed that ST-RNN outperformed the futuristic methods and can model the spatial and temporal contexts.

A classification approach for decision trees to predict the next place of mobile users was presented by [35]. As every client tends to have a broad pattern of behavior, therefore for each individual, the streamlining agent was executed to locate the best parameters combination. Consequently, the execution of this approach was appeared by the results of the examinations on the real-life dataset of 80 mobile clients presented by Nokia.

This research paper presents a process, which includes assembling information from literature and combining it uniquely with the feedback acquired from a few developers and contributors through emails. The surveys carried by the authors consider several algorithms. There are a number of algorithms that need to be evaluated similarly. We have taken into consideration a large pool of algorithms that none of the surveys has considered before this study. There is a gap in choosing trajectory prediction from the available literature. This study provides a platform where all vital parameters are aggregated in a single location.

## 4. Datasets

This section briefly describes the algorithms that are used by each study that are surveyed in this research article. Each algorithm is evaluated on a given dataset. These datasets are categorised into outdoor and indoor in addition to having a location component. Moreover, the type of dataset carries a certain weight-age in ranking as per our defined criteria. The multiple datasets along with brief discussion also gives a better insight to professionals.

### 4.1. American Time-Use Survey (ATUS)

ATUS (https://www.bls.gov/tus/) is a survey that provides the temporal information of people on various activities such as working, socialising, childcare, leisure, volunteering and household. ATUS is conducted by United States Census Bureau (USCB) and sponsored by the Bureau of Labor Statistics (BLS). The records are accumulated from year 2003 to 2016 from more than 180,000 meetings. The total size of the dataset is 150 Mega Bytes (MB). A comprehensively illustrative appraisal of how, where, and with whom Americans spend their time. It is the principle government survey giving data to the full extent of nonpublic works out, from childcare to volunteering, as given out by the American Time Use Survey (ATUS). A far-reaching extent of problems are utilized using ATUS data reports by researchers to contemplate; the records and information are accumulated from 2003 to 2016 from more than 180,000 meetings. The total size of the dataset is 150 (MB) and this information is propriety of U.S. Bureau of Labor Statistics and they have made it accessible to the general population.

### 4.2. Microsoft Multi-person Location Survey (MSMLS)

MSMLS (https://www.microsoft.com/en-us/research/publication/the-microsoft-multiperson-location-survey/) is a research project under which traveling patterns of general public are collected. Microsoft has provided GPS receivers to test subjects which were left in their cars for 2 weeks. The GPS data provides location coordinates along with time-stamps. The main aim is to assist drivers in better navigation to their destinations. This is an ongoing research venture to survey the travel examples of consistent individuals. Microsoft lent a GPS receiver to their volunteers, that can measure and storing time-stamped (latitudinal, longitudinal) information. The volunteers leave the GPS gadgets in their autos for around two weeks, it will record everywhere the car goes during that time by the GPS sensor. When these volunteers bring back the GPS sensors, the stored data is uploaded to servers of Microsoft. Research projects are being pursued by Microsoft to predict a driver’s destination and generating efficient driving directions. This is a proprietary dataset as they have not made it available for the public.

### 4.3. Phonetic

Phonetic Dataset (http://www.clres.com/) are sound recordings (approximately 70,000 in British English and 35,000 in American English) with dataset size of approximately 3.1 MB.

### 4.4. GeoLife

Microsoft GeoLife (https://www.microsoft.com/en-us/download/details.aspx?id=52367) is a project which collected 182 users GPS data for a period of three years (April 2007 to August 2012). A total distance of 1.2 million kilometers was measured in this project. The dataset consists of 17,621 trajectories that took a total duration of 48,000+ h. Different GPS loggers and GPS-phones were used to record these trajectories. Many users recorded outdoor trajectories of this dataset that not only comprised of normal routines such as going home and going to work but also included other activities such as entertainment, sports activities, shopping, sightseeing, hiking, dining, cycling etc.

### 4.5. CenceMe

CenceMe (http://cenceme.org/) project uses the standard sensor enabled mobiles phones for collecting social data in real time. For example, multiple activities such as walking, running and dancing were recorded. The size of this dataset is about 252 MB.

### 4.6. TIGER

The Topologically Integrated Geographic Encoding and Referencing (TIGER) (https://gisgeography.com/tiger-gis-data-topologically-integrated-geographic-encoding-referencing/) dataset contains spatial data such as railway lines, river, roads, legal, statistical geographic areas and hydrography.

### 4.7. Thomas Brinkhoff

Thomas Brinkhoff (https://iapg.jade-hs.de/personen/brinkhoff/generator/) is a network-based data generator of moving objects. It combines user-defined specifications for generating relevant datasets. Thomas Brinkhoff is used in multiple studies related to moving object analysis.

### 4.8. MIT Reality

MIT Reality (http://realitycommons.media.mit.edu/) project used 100 Nokia (6600 model) cell phones to record the data. The data comprised of calling records, bluetooth recordings, cell tower IDs, application usage and telephone state. The data was collected over the span of nine months. The dataset size is about 56 MB.

### 4.9. Augsburg Indoor Location Tracking Benchmarks

Augsburg Indoor Location Tracking Benchmarks (https://www.informatik.uni-augsburg.de/de/lehrstuehle/sik/publikationen) includes the movements of four persons through an office building. The movement data were collected from July 2003 till January 2004 at the fourth floor of the building of the Institute of Computer Science at the University of Augsburg. The Benchmarks data ranges from 101 to 448 different locations. They began predicting with a similar four test people in the fall semester of 2003 for more than five weeks. The fall semester data ranges from 432 to 982 different locations with a total size of 182KB.

## 5. Public and Propriety Datasets

The algorithms that are using public and propriety datasets are shown in Table 1. The datasets Uniform Resource Locators (URLs) are available mostly for public datasets as shown in Table 1, whereas N/A represents that no link is available. Furthermore the table also shows whether the data type is indoor or outdoor. This information can be useful for researchers and industry professionals.

**Figure 1:**
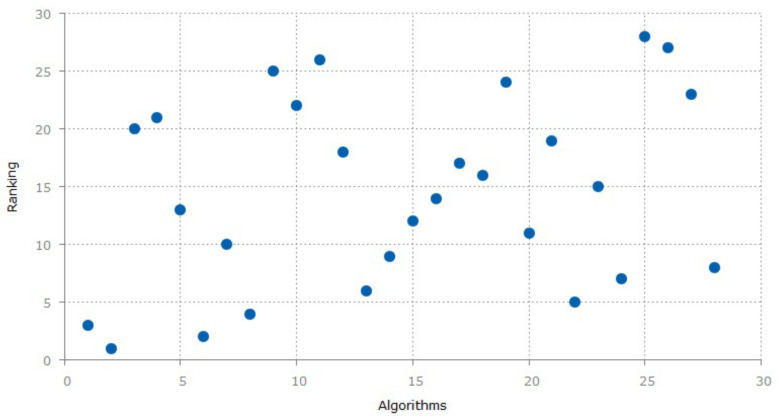
Ranks of Public and Propriety datasets. The algorithms on the *x*-axis show the ranking that were used by the papers. Each point on the *x*-axis depicts a study from Table 1.

Similarly, Figure 1 shows the ranking of public and propriety datasets. On *x*-axis, the algorithms are numbered (using serial number from each row of Table 1), while *y*-axis shows the ranking of the algorithms used in each paper that are being surveyed. The ranking criteria is described in Section 6.

## 6. Our Approach

This section describes our approach towards calculating ranking of algorithms based on number of criteria. Our approach focuses on the state-of-the-art, time and data efficient and accurate outdoor algorithm. One of the key observations was that few authors used multiple datasets to validate their approach towards trajectory prediction. We assigned more weight-age to multiple dataset used in various studies. GPS data in outdoor environment was given the most importance while ranking. Similarly, outdoor datasets in comparison to indoors were given more weight. Citation is another important aspect which is considered for ranking algorithms. The more the citations of a given algorithm, the more weight-age is assigned. This also shows more trust in the research community on a particular algorithm.

(6) $$R_{\left(S\right)}\;=\;A_{\left(C\right)}\;+\;Y_{\left(R\right)}\;+\;D_{\left(I\right)}\;+\;D_{\left(O\right)}\;+\;C_{\left(T\right)}\;$$

Ranking System R_(S)_ was calculated against the following criteria as shown in Equation (6), where A_(C)_ defines algorithm accuracy, Y_(R)_ is year of publication, D_(I)_ denotes indoor datasets, D_(O)_ refers to outdoor datasets and C_(T)_ is the number of citations as per Google Scholar. The higher the given criterion, the higher is the weight-age.

Furthermore, the years in which articles are published and the algorithms authors have used were added. The most recent algorithms were given the higher weightage so that the new accurate prediction algorithms that have less citations can also contribute to the overall ranking. This way the top new algorithms and the old best algorithms with large number of citations can qualify for results. Finally the top three algorithms from the survey were selected.

We further present algorithms information in form of multiple tables. These algorithms are classified into Markov model (Table 2), hidden Markov model (Table 3), T-pattern tree (Table 4), Bayesian network (Table 5) and distinct algorithms (Table 6). All these tables show the accuracy and citations of individual algorithms in the respective categories.

Table 1 shows the studies that are surveyed in this paper. The serial number of each row is later used in the ranking. Table 1 also shows which dataset was used for each algorithm by the authors, and also whether the dataset was publicly available or if it was a propriety dataset. Furthermore, links for data download are available for the public datasets while propriety dataset are restricted. The last column depicts if the algorithm was tested on indoor or outdoor datasets. Table 2 shows the papers that have used the Markov model for predicting locations. The data shown in table is our criteria. i.e., the year the paper was published, the accuracy of the predicted algorithms, the datasets used by the algorithms, the citations and indoors or outdoors datasets. Similarly, Table 3 shows details of algorithms based on the hidden Markov model. Table 4 shows the T-pattern tree algorithm details. On the other hand, Table 5 shows the Bayesian network details while the distinct algorithm details are shown in Table 6. These distinct algorithms are not previously categorized. This information is then fed to the criteria’s logic to find the top three algorithms.

**Figure 2:**
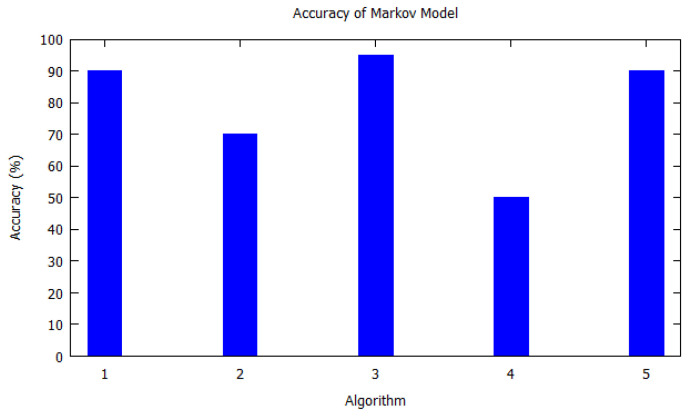
Accuracy of Markov model. Markov model as per Table 2 is shown with *X*-axis depicting the algorithms the paper used, i.e., the serial number from Table 2. *Y*-axis shows the accuracy of the algorithm that was used by the authors for that study.

**Figure 3:**
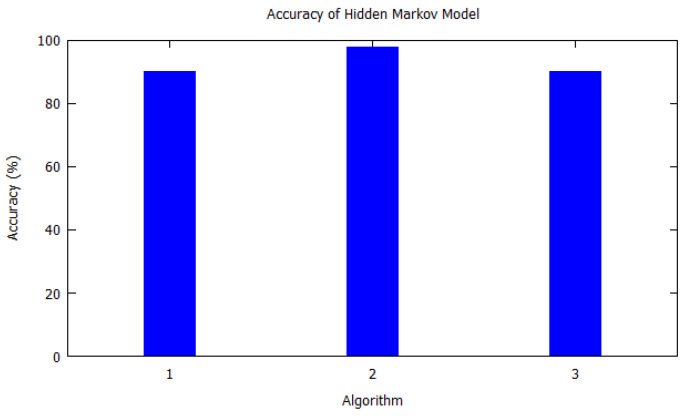
Accuracy of hidden Markov model. Hidden Markov model as per Table 3 is shown with *X*-axis depicting the algorithms the paper used, i.e., the serial number from Table 3. *Y*-axis shows the accuracy of the algorithm that was used by the authors for that study.

**Figure 4:**
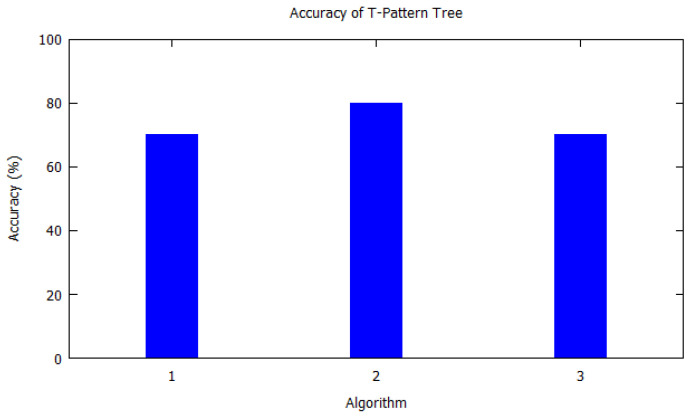
Accuracy of T-pattern tree. T-pattern tree from Table 4 is shown with *X*-axis depicting the algorithms the paper used, i.e., the serial number from Table 4. *Y*-axis shows the accuracy of the algorithm that was used by the authors for that study.

**Figure 5:**
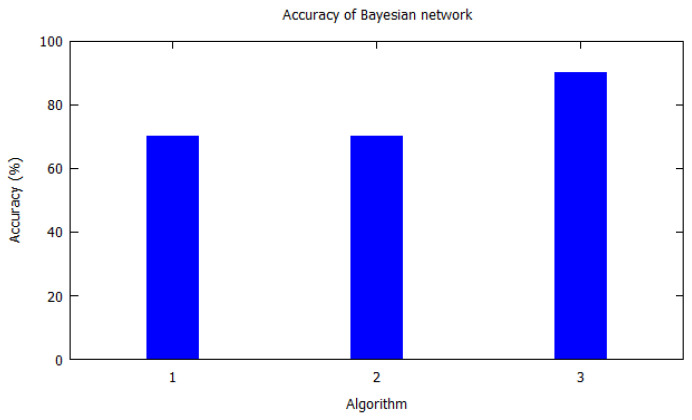
Accuracy of Bayesian network. Bayesian network from Table 5 is shown with *X*-axis depicting the algorithms the paper used, i.e., the serial number from Table 5. *Y*-axis shows the accuracy of the algorithm that was used by the authors for that study.

**Figure 6:**
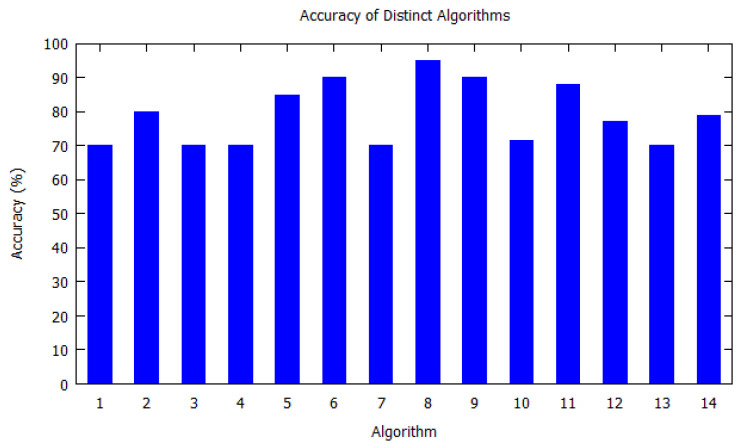
Accuracy of Distinct Algorithms. Distinct Algorithms from Table 6 is shown with *X*-axis depicting the algorithms the paper used, i.e., the serial number from Table 6. *Y*-axis shows the accuracy of the algorithm that was used by the authors for that study.

Similarly, bar charts are drawn for improved visual understanding on the accuracy of algorithms. For each table corresponding bar graphs are drawn. In Figure 2, Markov model from Table 2 is shown with *X*-axis depicting the algorithms used, i.e., the serial number as per Table 2. Similarly, the *Y*-axis shows the accuracy of the algorithm that was used by the authors for that study. In Figure 3, Hidden Markov model is visualized as per Table 3. Figure 4 illustrates the accuracy of algorithms used by the papers in Table 4 for the T-pattern tree. Similarly, Figure 5 corresponds to Table 5 for Bayesian network. The distinct algorithms can be seen in Figure 6. Most of the algorithms have achieved accuracy more than 70%.

## 7. Results and Discussion

Based on the proposed methodology, three of the best and unique algorithms were selected. Keeping in mind that if two algorithms made it to the top, one of them was rejected as we wanted to propose three of the best as well as unique algorithms for the survey. Table 7 highlights the three best algorithms.

A brief discussion of these algorithms is as follows:

Next Place, which qualified at the top as per our algorithm, is a nonlinear time analysis technique was used to predict single user’s most important location. The author used four different datasets and achieved up to 90% probability for users next location along with performance increment of at least 50%. This technique uses a new predictive framework which was developed for a spatiotemporal viewpoint. This does not consider the transitional locations that comes in between the most significant locations of the user. The algorithm predicts the duration of visit, the residence and arrival time of the user. The random behavior which is normally considered as an anomaly is also captured by this approach. The author described an effective technique to predict the user significant location. Once the significant locations are identified then the residence time at those locations are predicted and the amount of time for next visit. NextPlace also predict further in the future which existing models are unable to do. Furthermore, four human movement datasets (which is one of the criteria in our approach) were utilized for this research. These datasets considered total number of attributes including number of users, number of visits, number of significant places, average number of significant places per user, average number of visits per users, average residence time, total trace length, and average time spent by each user in significant places.

In the Markov model, the authors used the algorithm for predicting the nearest route taken by the vehicle driver. Markov Model was used to make probable locations by analyzing the drivers recent routes. The data is analyzed over a long period of time from the GPS history of the vehicle. The algorithm takes account of the discrete road segments. Here, the authors predicted the next road segments with 90% of accuracy. The application of this algorithm is traffic disruptions to drivers and automation of vehicle behaviours using forecasting. The Markov model was the top prediction algorithm as per our criteria and due to its simplicity, effectiveness and accurate prediction.

The hidden Markov model proposes a visit-history-based activity prediction algorithm for services of activity-aware mobile in smart cities named Agatha. The algorithm uses region-based activities, the likelihood that where you have been so far affects what you will do immediately. The casualties are inherent in common daily activities that regularly reveal a development pattern. The model gets such development causalities similarly as an activity course of action plans from supposed ground-breaking settings, for instance, visit place, visit time, term, and transportation mode. This algorithm efficiently deals with the complexity of learning new causalities between various diverse convincing settings. The prediction model was evaluated using the American Time-Use Audit (ATUS) dataset that fuses more than 10,000 individuals’ locations and activity history. The evaluation results show that it can envision customers’ relied upon practices with up to 90% of accuracy for the top three activities. The hidden Markov model also made it to top algorithms as per our methodology.

## 8. Conclusions

This article provides analysis of 28 algorithms that we surveyed in the area of trajectory location prediction. This survey was carried out to provide guidelines for professionals (researchers and developers) to select best suited algorithms for their needs which was otherwise missing in the literature.

We applied a simple yet effective approach to come up with a ranking of available algorithms. Firstly, an extensive literature review was done to choose candidate algorithms covering the domain of GPS trajectory prediction. In order to preform ranking multiple criteria was selected such as dataset type (public and/or propriety datasets), the number of citations a particular algorithm received over a number of years, and use of outdoor and/or indoor datasets. Moreover, while ranking the algorithms, other parameters are also considered such as accuracy (performance) and ease of use. The results gives top three best algorithms which are selected and recommended as part of this study. These include NextPlace, Markov Model, and Hidden Markov Model. These algorithms when studied are quite simple yet effective for trajectory prediction. Trajectory research has gained a lot of pace in last decade. There are many use cases which can be effectively explored and implemented. For example, health care engineering, transportation, socialization, artificial intelligence, surveillance, and indoor navigation’s.

As part of future work, we plan to add more algorithms which can further strengthen our ranking mechanism. Furthermore, we will consider other factors such as algorithm complexity, application use, bench marking, context awareness, prediction robustness, and integration [45] for decision-making in electronic health care applications.

## Figures and Tables

**Table 1:** Public and Proprietary Datasets.

S No.	Paper	Dataset	Dataset Type	Link	Indoor/Outdoor
1	[9]	American Time Use Survey (ATUS)	Public	https://www.bls.gov/tus/data.htm	Both
2	[7]	GPS Cabspotting	Public	https://stamen.com/work/cabspotting/	Both
3	[36]	GPS Dataset	Propriety	N/A	Outdoor
4	[25]	Mobile Data Challenge (MDC) Dataset	Propriety	https://www.idiap.ch/dataset/mdc	Outdoor
5	[12]	Vehicle GPS Dataset	Propriety	N/A	Outdoor
6	[8]	GPS Dataset	Propriety	N/A	Outdoor
7	[37]	Vehicle GPS Dataset	Propriety	N/A	Outdoor
8	[30]	GPS Dataset	Propriety	N/A	Outdoor
9	[14]	Movement data	Propriety	N/A	Outdoor
10	[15]	T.Brinkoff	Public	https://iapg.jade-hs.de/personen/brinkhoff/generator/	Outdoor
11	[16]	Moving Objects Databases	Propriety	N/A	Outdoor
12	[38]	GPS Dataset	Propriety	N/A	Outdoor
13	[33]	Phonetic, GeoLife	Public	http://www.clres.com/phonetic.html https://www.microsoft.com/en-us/download/details.aspx?id=52367	Outdoor
14	[39]	GPS driving data	Propriety	N/A	Outdoor
15	[17]	MIT Reality Dataset	Public	http://realitycommons.media.mit.edurealitymining.html	Outdoor
16	[18]	Synthetic data	Propriety	N/A	Outdoor
17	[40]	TIGER	Public	http://spatialhadoop.cs.umn.edu/datasets.html	Outdoor
18	[34]	Mobility Data	Propriety	N/A	Both
19	[41]	Automatic Vehicle Location (AVL) System	Public	http://ieeexplore.ieee.org/document/5174540/?reload=true	Outdoor
20	[31]	CDR dataset	Propriety	N/A	Both
21	[35]	Nokia Real life Dataset	Public	https://www.idiap.ch/dataset/mdc	Both
22	[42]	GPS Sensors Data	Public	N/A	Outdoor
23	[43]	Smartphones Mobility Data	Propriety	N/A	Both
24	[32]	Smartphone logs	Propriety	N/A	Both
25	[6]	Augsburg Indoor Location Tracking Benchmarks	Public	https://www.informatik.uni-augsburg.de/en/chairs/sik/research/finished/ailtbenchmarks/	Indoor
26	[19]	Augsburg Indoor Location Tracking Benchmarks	Public	https://www.informatik.uni-augsburg.de/en/chairs/sik/research/finished/ailtbenchmarks/	Indoor
27	[44]	Mobility Dataset	Propriety	N/A	Both
28	[20]	MIT Reality Dataset	Public	http://realitycommons.media.mit.edu/realitymining.html	Both

**Table 2:** Markov model.

S No.	Year	Algorithms	Author	Accuracy	Dataset	Indoor/Outdoor	Citations
1	2016	Markov model	[8]	90%	GPS data from volunteer drivers in Microsoft Multiperson Location Survey (MSMLS)	Outdoors	171
2	2003	Markov model	[30]	70%	Automatic clusters GPS data taken over an extended period of time	Outdoors	1383
3	2012	Markov model	[33]	95%	Three datasets: Phonetic, Geolife, synthetic dataset	Outdoors	396
4	2012	Nine different baseline models	[25]	50%	Mobile dataset provided by Nokia Mobile Data Challenge which contains 80 users over one year of time	Outdoors	135
5	2011	NextPlace, derived from Markov model	[7]	90%	Four datasets: two GPS-based (CenceMe) and two registration patterns of Wi-Fi access points	Both	340

**Table 3:** Hidden Markov model.

S No.	Year	Algorithms	Author	Accuracy	Dataset	Indoor/Outdoor	Citations
1.	2015	HMM	[9]	90%	American Time-Use Survey (ATUS) dataset	Both	5
2.	2006	HMM	[36]	98%	Low-cost GPS unit	Outdoor	227
3.	2016	HMM	[32]	90%	Real everyday life datasets collected from 10 persons for six months	Both	50

**Table 4:** T-pattern tree.

S No.	Year	Algorithms	Author	Accuracy	Dataset	Indoor/Outdoor	Citations
1	2009	T-pattern tree	[12]	70%	Dataset of 17,000 cars equipped with GPS	Outdoors	617
2	2012	Decision tree	[35]	80%	Real-life dataset provided by Nokia	Both	31
3	2004	Spatiotemporal prediction tree	[40]	70%	Real point dataset (Tiger)	Outdoors	354

**Table 5:** Bayesian network.

S No.	Year	Algorithms	Author	Accuracy	Dataset	Indoor/Outdoor	Citations
1	2006	Predestination	[37]	70%	Microsoft Multiperson Location Survey (MSMLS)	Outdoors	586
2	2016	Enhanced Bayes predictor	[31]	70%	CDR dataset with more than 3.5 billion calls	Both	3
3	2005	Transition matrix (TM)	[34]	90%	Personal Communication Systems	Both	394

**Table 6:** Distinct algorithms.

S No.	Year	Algorithms	Author	Accuracy	Dataset	Indoor/Outdoor	Citations
1	2006	Apriori-Traj Algorithm	[14]	70%	Network-based generator of moving objects	Outdoors	96
2	2007	Traj-Prefix-Span Algorithm	[15]	80%	Synthetic datasets by T.Brinkhoff	Outdoor	209
3	2003	Query-Triggered Revision with Query Relaxation	[16]	70%	Moving objects database	Outdoor	58
4	2008	Hybrid Genetic Algorithm	[38]	70%	GPS dataset	Outdoors	363
5	2008	Route Prediction	[39]	85%	GPS data from 252 drivers	Outdoor	278
6.	2011	Seman Predict	[17]	90%	MIT Reality Dataset	Outdoor	304
7.	2008	The Hybrid Prediction Model	[18]	70%	4 synthetic datasets	Outdoor	327
8.	2003	Time of Arrival Prediction	[41]	95%	Automatic vehicle location (AVL) systems	Outdoors	122
9.	2004	State Predictor Method	[19]	90%	Augsburg Indoor Location Tracking Benchmarks	Indoors	15
10.	2016	TLP (Trajectory similarity-based approach for location prediction)	[20]	71.60%	MIT Reality Mining Dataset	Both	68
11.	2016	Contextual Location Prediction	[42]	88%	GPS, GSM and Wi-Fi data	Outdoors	6
12.	2012	Smartphone-based Mobility Prediction	[43]	77%	153 mobile users data	Both	130
13.	2017	Gaussian Process Regression	[44]	70%	Large-scale mobility datasets at a city level	Both	12
14.	2005	Elman Net, MLP, Bayesian Network, State Predictor, Markov Predictor	[6]	79%	Augsburg Indoor Location Tracking Benchmarks	Indoors	70

**Table 7:** Top Algorithms.

S. No	Algorithm	Datasets	Paper
1	NextPlace	Four datasets: Two GPS-based (CenceMe) and two registration patterns of Wi-Fi access points	[7]
2	Markov model	GPS dataset	[8]
3	Hidden Markov model (HMM)	American Time-Use Survey (ATUS) dataset	[9]
